# Exploring Clinician Experiences With a Digital Platform Supporting Orthopedic Care That Integrates Patient-Generated Health Data: Qualitative Study of Early Users

**DOI:** 10.2196/65216

**Published:** 2025-08-29

**Authors:** Dmitri S Katz, Daniel Gooch, Linda Price, Irum Rauf, Oliver Pearce, Blaine Price

**Affiliations:** 1 School of Computing and Communications, The Open University, Walton Hall, Milton Keynes, MK7 6AA, United Kingdom, 44 1908858234; 2 Trauma and Orthopaedics, Milton Keynes University Hospital, Milton Keynes, United Kingdom

**Keywords:** patient-generated health data, PGHD, orthopedic care, digital health, remote monitoring, patient-centered care, clinician workload, patient engagement, health informatics, orthopedics, quality of life, physiotherapists, surgeons

## Abstract

**Background:**

Digital care platforms that integrate patient-generated health data (PGHD) alongside education and communication tools have been recognized as potential instruments in transforming health care from clinician-centered to a more patient-centered approach. This transformation is driven by the potential of PGHD to provide deeper insights into patients’ conditions, facilitate personalized care, improve patient quality of life, reduce inefficiencies in data collection, and empower patients. Yet, actual implementation within clinical settings is still at early stages; therefore, impacts on clinical care remain limited.

**Objective:**

This study sought to explore the benefits, challenges, and opportunities of integrating PGHD into orthopedic care by analyzing the reflections of early adopter surgeons and physiotherapists who have used a digital care management platform.

**Methods:**

This qualitative study used thematic analysis of interviews conducted with surgeons and physiotherapists (n=9) from a clinical unit that was among the first to trial “mymobility,” an industry-produced software platform (Zimmer Biomet). The participants were recruited using snowball sampling, and interviews were conducted from June to July 2022. The interviews focused on work practices, use of digital tools, experiences with PGHD, and experiences with the mymobility software. Thematic analysis was conducted using NVivo software (QSR International Pty Ltd), focusing on identifying key themes and insights.

**Results:**

The study identified several benefits of integrating PGHD into orthopedic care, including improved patient education, enhanced communication and assessment, and increased patient motivation and adherence. However, several challenges were also noted, such as increased clinician workload, questionable data utility, lack of patient centricity, and inability to tailor software to clinical contexts. Suggested opportunities included improving dashboard design, personalizing physiotherapy, and using collected data for improving clinical care.

**Conclusions:**

The integration of PGHD into orthopedic care shows promise, largely in areas suggested by the literature. However, significant challenges remain. Future research should focus on addressing solvable challenges, such as improving software user interface design and functionality, while embracing the possibility that some challenges lack clear solutions and will likely require careful balancing of design tensions. The findings highlight the need for ongoing development and refinement of PGHD-inclusive systems to better support clinical practice and patient outcomes.

## Introduction

### Overview

There are frequent calls within academic literature and popular media to transform health care systems from the traditional “clinician-centered” model to a more “patient-centered” approach, an approach characterized by shared clinical decision-making and increased patient autonomy [1] while also helping to reduce costs through coordinated and accessible personalized services [2]. An important and often asserted mechanism for this transition is increasing the use of patient-generated health data (PGHD) within clinical care. PGHD are information collected by individuals (and informal caregivers) on aspects such as their treatment records, symptoms, biometric data, and lifestyle preferences [3]. The use of PGHD has been promoted for various reasons: providing deeper insight into a patient’s condition [4], facilitating personalization of care [5], improving patient quality of life [6], reducing inefficiencies and insufficiencies in data collected solely at clinical visits [7], reducing retrospective recall bias [8], and empowering patients by helping them to understand their progress [9].

Using PGHD has become increasingly feasible, as technologies for self-tracking of health and wellness data have become affordable and culturally acceptable to consumers [10]. The ease of data collection for patients and ubiquity of these devices are informally influencing clinical interactions, as patients increasingly present their self-collected data during clinical visits [5], and recent studies suggest that clinicians are increasingly open to using PGHD at least as supplementary data within clinical workflows [11] [4].

Despite the potential benefits, there remain extensive known barriers to implementing PGHD within clinical care. For example, West et al [5] identified the nature of the data (structure, completeness, reliability, lack of context, and relevance), selective disclosure, clinician suspicions of underlying patient psychiatric disorders, insufficient clinician time, insufficient clinician expertise, clinician overload, interoperability, and negative impacts to workflow. Other notable barriers include clinician reluctance to assume responsibility for motivating patients to collect data [4], clinician concerns that reliance on PGHD could exacerbate health disparities [6], negative impacts on patient-clinician relationships [12], and resistance to change within clinical culture [7]. Given such diverse challenges, it is perhaps unsurprising that formal integration of PGHD within clinical practice remains limited [13]. Therefore, there remains a lack of knowledge on diverse PGHD-related topics such as how to use PGHD to improve health care outcomes [3], impact on patient-clinician communication within surgical domains [12], best practices to incorporate into clinical workflows [14], and impacts in supporting clinical practice [11].

This study sought to understand work practices, acceptance of digital tools, and attitudes toward PGHD as part of a broader care management platform to gain insights that could guide the design of digital systems that incorporate PGHD alongside other patient support functionalities in orthopedic care. To this end, we conducted interviews with surgeons and physiotherapists who were members of an “early adopter” orthopedic unit that had (1) been given the opportunity to use an early release of industry-produced patient journey software that offered various tools for interacting with patients and their data and (2) participated over several years in the development and deployment of a novel device to enable orthopedic patients to self-collect pain data [15]. Focusing on this context foregrounds real-world exposure and engagement with PGHD, with the reported experiences helping to fill current gaps in knowledge in the clinical use of PGHD, guiding future research on the use of these data in a clinical context.

### Background

Total knee arthroplasty (TKA) and total hip arthroplasty (THA) are common elective surgeries for older adults, where an original joint damaged by arthritis or trauma is replaced with metal and plastic implants. Due to high cumulative expenses resulting from this increasingly common procedure, TKA or THA operation is an important target for optimizing care [16]. To this end, fast-track programs that seek to reduce patient time in the hospital have become standard, successfully reducing costly hospital stays without impairing outcomes or increasing readmissions [17]. However, despite the successes of this approach, there remain opportunities for improvement that could potentially be supported with increased use of PGHD. For example, patients who have had a TKA or THA operation frequently have risk factors that can be related to readmission (eg, obesity, cardiovascular disease, diabetes, and lifestyle factors) [16]. Yet, current clinical systems often fail to consider what occurs outside the boundaries of the health care system [18]. This lack of vital information about patients’ comorbidities and lifestyle can hinder timely decisions about surgical scheduling and personalizing patient care, and there is also an urgent need to increase proactive care to enhance patient satisfaction, promote long-term recovery, and reduce unnecessary hospital stays. Given the importance of events occurring outside of the clinical setting for improving outcomes, the importance of engaging patients in collaborative care, and the potential for nonclinical data to inform policies that could improve outcomes, TKA or THA appears as a promising domain for increased PGHD integration.

### PGHD and THA or TKA

The adoption and affordability of wearable devices have increased the feasibility of integrating PGHD into THA or TKA care, and there have been numerous studies exploring potential benefits and limitations. Toogood et al [19] used ankle-worn Fitbit step counters to track daily steps in patients who have had their THA operation. They found this method feasible and found that by differentiating activity levels based on factors like age, BMI, surgical approach, and discharge destination, it might be possible to identify slower recovery cases that could benefit from interventions. However, other studies cautioned that while tracking was technically feasible, the resulting data had practical limitations. For example, Crizer et al [20] studied using step counts to monitor preoperative and postoperative recovery but found a weak correlation with patient-reported outcomes. The authors suggested that combining objective measures like step counts with patient-reported outcomes would provide a more comprehensive assessment. Similarly, Bendich et al [21] examined data from wearable sensors worn by patients both before and after total joint surgery. They reported that alterations in sensor data 6 weeks after surgery correlated closely with patients’ reported improvements in well-being; however, the raw sensor data did not directly correspond with patients’ self-reported feelings, suggesting that to effectively predict postsurgery outcomes using sensors, it is essential to analyze how the data evolve over time rather than solely focusing on absolute values. These papers highlight that it is not sufficient to aggregate and report data to clinicians, but rather that meaningful interpretation and context are essential. We found numerous papers that applied tracking devices to support patient monitoring outside the clinical environment. However, we found little research on the reflections of orthopedic clinicians on the use of PGHD and more specifically their experiences with PGHD-inclusive clinical software. In addition, relatively few studies on PGHD integration have examined the use of patient-generated data from the clinician’s perspective rather than the patient’s perspective [5], which is important, as clinician engagement has previously been recognized as a significant challenge for integrating patient self-reporting systems into clinical practice [7]. This paper aims to fill these gaps through exploring clinicians’ real-world experiences with the benefits and challenges of integrating PGHD into TKA or THA workflows and through analysis identifying opportunities to improve these systems.

## Methods

### Ethical Considerations

Ethics clearance was granted through the Open University Human Research Ethics board (HREC/3776/Mehta). Preconditions for participation were professional capability in either of the 2 selected clinical roles, experience with TKA or THA, and spoken English. All participants were employed in these roles in the United Kingdom. Participants were assured anonymity. Prior to the interview, participants received an email confirmation of the time and date, as well as receiving a copy of the questions and a consent form that was returned by email. Participation was voluntary, and all participants were informed of their right to withdraw at any time. The interviews lasted from 30 to 60 minutes and were conducted by the same member of the research team (LP). Participants were offered either face-to-face or digital interviews to accommodate their clinical schedules. The interviewees’ responses were anonymized according to the ethics committee procedure. Each institution’s data protection policy was adhered to, ensuring that data were confidentially stored. All transcripts were transcribed and stored anonymously, and the data were stored on a secure system only accessible to the research team. Interviewees received approximately US $34 honorarium for taking part in the interviews.

### Study Design

This study was designed as a formative qualitative investigation to surface early insights from clinicians engaging with PGHD-inclusive digital tools. It was not intended to quantify impact or compare outcomes but rather to inform future system design and identify areas warranting further evaluation through quantitative or mixed methods research.

We chose to interview 2 essential and complementary clinical roles in the TKA or THA domain: orthopedic surgeons who are central to the procedure [22] and physiotherapists whose work has been shown to improve postsurgical recovery [23]. These 2 clinical roles are directly involved in interpreting and responding to PGHD within the mymobility platform (Zimmer Biomet). Surgeons reviewed data to monitor recovery progress and inform follow-up care, while physiotherapists engaged with exercise adherence and symptom reports to adjust rehabilitation plans. Their combined perspectives were essential for understanding how PGHD are integrated into orthopedic workflows. Participants were selected using purposive sampling to capture a range of clinical and implementation perspectives from early adopters of the platform. Recruitment occurred during the COVID-19 pandemic, which introduced additional constraints such as staff unavailability, clinical backlogs, and limited access to nonurgent personnel. These conditions restricted the expansion of the sample. Our team was composed of human-computer interaction professionals (an interdisciplinary field that studies how people interact with digital technologies, with the goal of designing systems that are usable, effective, and aligned with users’ needs) and clinicians, a pairing recommended by Cheng et al [24]. Our team also included a change management specialist with experience in digital system implementation. This team member supported interview design and interpretation by contributing expertise on organizational readiness, clinician engagement, and adoption patterns as well as conducting interviews. This team member’s perspective helped to frame questions around perceived barriers and enablers in relation to system-wide change processes. Our research was also guided by a sociotechnical systems design (STSD) approach, which seeks to balance human, social, organizational, and technical requirements. This perspective recognizes that digital systems—particularly those introduced into complex clinical environments—often fail when they overlook the interdependent and often hidden dynamics of the social and organizational contexts in which they are deployed [25]. The STSD perspective was used as an interpretive lens for understanding clinician experiences and guiding the interpretation of clinician reflections in relation to their work systems, roles, and digital interactions. It was not applied as a formal evaluative framework, and no inferential claims are made. Given the qualitative nature and small sample size of this study, STSD served to sensitize analysis to the dynamic interplay between human, technical, and organizational factors in digital orthopedic care. Our research group has been collaborating with clinicians within this group since 2016 on the use of tracking devices to assess compliance [26] as well as research and design of tangible devices for patient pain logging [15,27,28]. In addition, members of this unit are participating in early testing of “mymobility,” an industry-produced orthopedic care software platform that integrates diverse PGHD from Apple Watch and iPhone into clinical dashboards. These interviews were conducted from June to July 2022, a period when, due to the pandemic, many typically in-person services had been replaced with app-based communication, which by necessity gave clinicians additional exposure to using health-relevant data collected by patients. Drawing on our ongoing relationship with the orthopedic center at Milton Keynes University Hospital, we used snowball sampling to recruit 4 orthopedic surgeons (1 female and 3 male) and 5 physiotherapists (4 female and 1 male; Table 1). All had previous experience with TKA or THA procedures, with experience ranging from 1.5 to 25 years. Clinicians had varying levels of exposure working with PGHD at Milton Keynes University Hospital. While we did not track or analyze the number of patients whose PGHD were reviewed by each participant, all interviewees were directly involved in the early use of the digital platform in clinical care. Participants routinely engaged with patients contributing PGHD as part of their roles, with varying degrees of exposure depending on their clinical responsibilities. The goal of this study was to explore clinicians’ perspectives on PGHD integration, not to quantify its use across patient populations. In the reporting on interviews, S1-4 will be used for surgeons and P1-5 for physiotherapists.

**Table 1.** Demographics of participants.

Participant	Age (years)	Sex	Ethnicity	Role	Experience (years)
Surgeon 1	56	Male	Asian	Associated specialist	25
Surgeon 2	46	Male	Asian	Consultant orthopedic surgeon	25
Surgeon 3	45	Male	Asian	Trauma and orthopedic consultant, hip and knee	25
Surgeon 4	53	Female	White	Consultant orthopedic and trauma surgeon	15
Physiotherapist 1	25	Female	White	Rotational physiotherapist	1.5
Physiotherapist 2	56	Female	White	Senior team lead physio in trauma and orthopedics	32
Physiotherapist 3	46	Female	White	Physiotherapist on trauma and orthopedics	12
Physiotherapist 4	29	Male	White	Musculoskeletal and orthopedic outreach physiotherapist	4
Physiotherapist 5	32	Female	White	Specialist musculoskeletal physiotherapist	3

**Figure 1.**
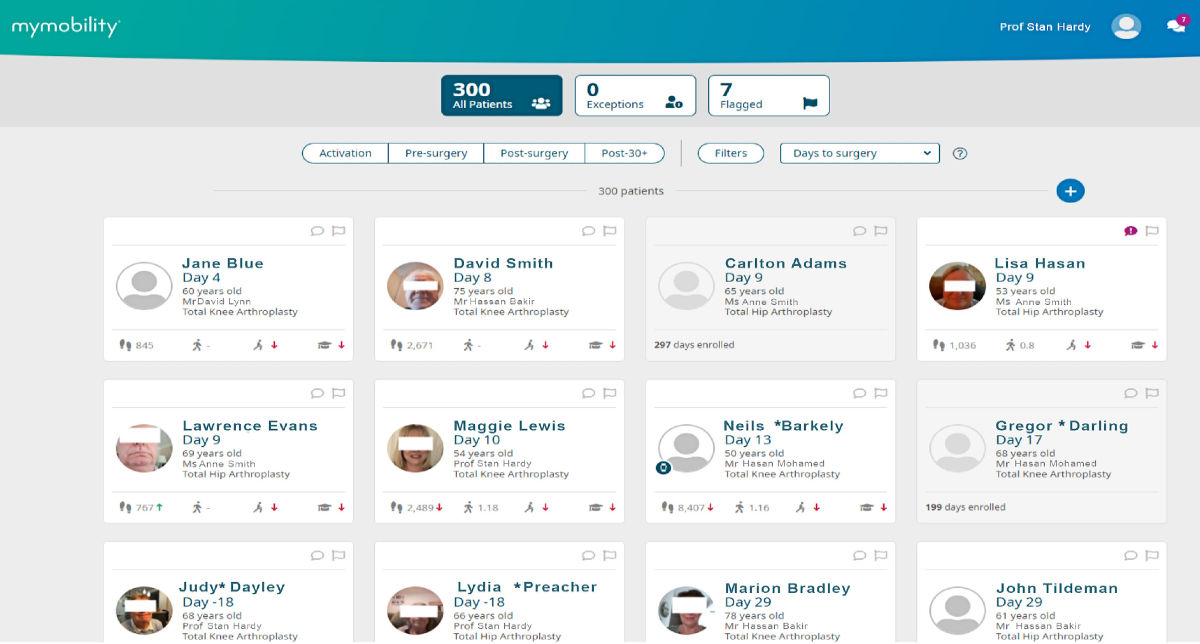
Patient overview.

Questions were based on our literature review and previous experience researching the requirements and design of digital systems. The interview questions were piloted and iterated with an advising senior orthopedic surgeon and a physiotherapist. The questions for the semistructured interviews were structured within four sections: (1) current work practices, (2) use of digital tools, (3) experiences with PGHD, and (4) experiences with the mymobility software platform. For the last set of questions, participants were provided with anonymized screenshots of PGHD from the mymobility app for their reference (Figures1  2).

**Figure 2.**
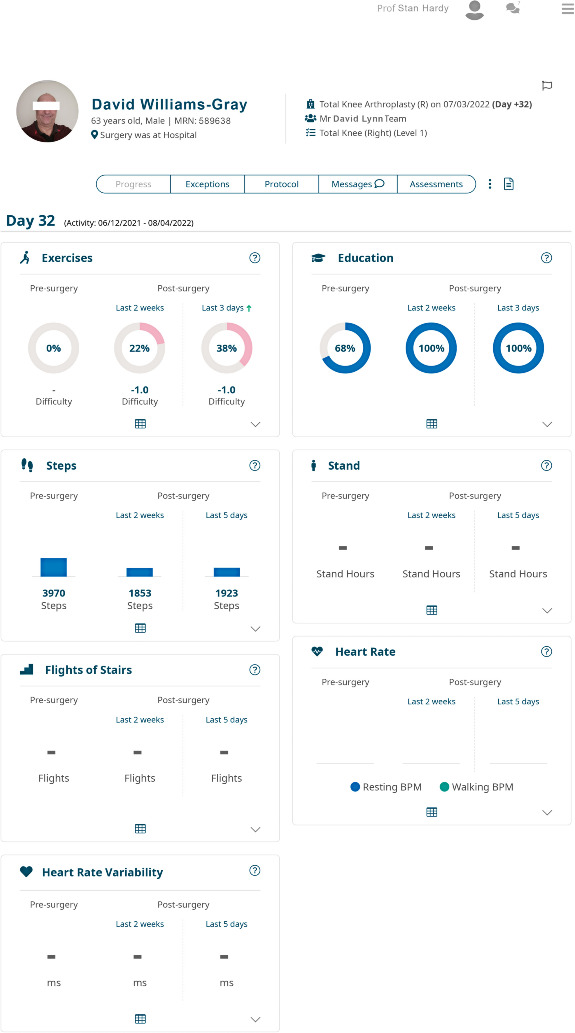
Patient progress.

### mymobility Software

mymobility [29] is a digital care management platform tailored for orthopedic patients, supporting a variety of procedures through care plans for knee, hip, shoulder, and spine surgeries. This platform seeks to facilitate communication and data sharing between patients and health care providers throughout the surgical process. Key features include:

- Patient engagement and compliance: The app seeks to foster patients’ engagement in their surgical journey by providing educational materials, procedural information, and personalized exercise plans.
- Data monitoring and insights: Through automated remote monitoring with Apple Watch and iPhone and patient-reported outcomes, the app tracks patient progress, allowing patients’ and clinicians’ dashboards to view collected data.
- Care team management: Through a unified platform for digital care management and telemedicine, the app seeks to support communication and coordination among patients and care team members.

### Data Analysis

The anonymized transcribed interviews were analyzed using reflexive thematic analysis (RTA), a qualitative approach that emphasizes the researcher’s active role in constructing meaning and identifying patterns across data [30,31]. RTA was selected for its suitability in capturing subjective clinician perspectives without imposing a fixed coding frame, aligning with our study’s exploratory and practice-based context. We adopted a realist stance—seeking to report participants’ experiences and meanings—while using an inductive, data-driven approach without applying a pre-existing theory or model. The analysis followed Braun and Clarke’s [30,31] 6-phase process: familiarization, coding, theme development, theme review, and final definition and naming.

Although the sample was limited (n=9), this reflects the formative nature of the study and recruitment constraints within a single National Health Service site during ongoing pandemic restrictions. While no formal assessment of data saturation was conducted, thematic redundancy across participants suggested that core experiential patterns were robustly captured. Consistent with Braun and Clarke’s [30,31] critique of saturation as a criterion borrowed from grounded theory, we recognized that RTA does not seek conceptual closure but rather embraces interpretative plurality and the coconstruction of meaning between researchers and data.

Analysis was conducted with NVivo software (version 12; QSR International Pty Ltd). The interviews were audio-recorded and transcribed. The answers were then collected in subcategories using open coding. Finally, similar open codes were grouped together and discussed in frequent subsidiary meetings with team members. While not all software functions within the mymobility platform are exclusively PGHD-related (education and messaging), and as the software integrates PGHD as a core functionality that can influence these interactions, and as our approach was not sufficiently fine-grained to distinguish between the impact and interactions of each of the features, we report on clinician reflections on these aspects as well.

## Results

### Benefits

#### Overview

**Figure 3.**
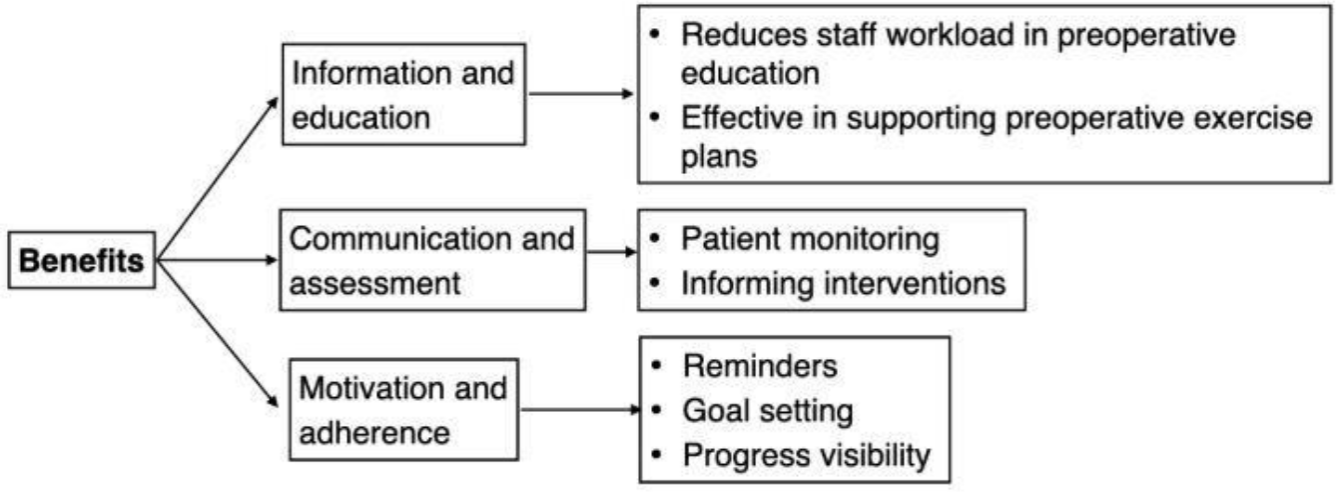
Benefits.

The mymobility app demonstrated benefits in patient information and education, communication and assessment, and patient motivation and adherence (Figure 3). Participants noted that the app reduced staff workload by providing preoperative information and exercise plans, ensuring that patients were well-prepared for surgery. Remote monitoring capabilities offered insights into patient activities and adherence, aiding clinical decisions and interventions. Additionally, features like reminders and goal setting improved patient adherence to clinical protocols.

#### Information or Education

Participants viewed the app as a valuable tool for reducing staff workload related to preoperative education. Several noted that it helped patients access key information and prepare for surgery independently. One physiotherapist explained that patients “get all the information ... we normally spend a lot of time going through with them,” giving them “an idea of what they’re getting themselves into” (Physiotherapist 3). Another remarked that patients find the preoperative education “very beneficial” (Physiotherapist 5). The app also supported home-based exercise routines, with participants noting that the protocol-based rehabilitation content was “absolutely fine prior to the operation” (Physiotherapist 4) and that it allowed patients to better “understand the exercises” (Surgeon 1).

#### Communication and Assessment

Clinicians consistently valued the app’s capacity to monitor patients remotely, particularly in terms of activity tracking, exercise adherence, and recovery progression. For example, one physiotherapist highlighted how access to pre-operative step counts offered a useful baseline for recovery expectations: “How active they are pre-op will influence their speed of recovery after op” (Physiotherapist 1). Another clinician appreciated the insight into adherence and difficulty with exercises, noting that the app enabled them to “see what exercises they’re doing ... and how well they’re adhering to it” (Physiotherapist 4).

Several participants described the platform as offering an unusually comprehensive overview. One surgeon described it as “brilliant ... it’s even doing your gait analysis ... the data we’re capturing, it’s unbelievable” (Surgeon 3). These data were also used to assess postoperative recovery patterns, helping clinicians gauge improvements or asymmetries over time: “It’s quite beneficial to give us a bit more of a guide as to how much load they’re putting through their operated limb” (Physiotherapist 5).

Beyond passive monitoring, participants emphasized the potential for the system to inform real-time interventions. One surgeon noted that the data made it easier to detect when patients were deviating from expected progress and intervene earlier if needed (Surgeon 1), while a physiotherapist described how being able to see prior prescribed exercises allowed for better continuity of care and responsive treatment adjustments (Physiotherapist 3). Another surgeon discussed how longitudinal data helped prioritize surgical scheduling for patients with deteriorating function: “We go through the app ... and we are able to assess that they need to be prioritized” (Surgeon 1).

#### Patient Motivation or Adherence

Participants valued the app’s features that supported patient motivation and adherence to rehabilitation protocols. Automated reminders were seen as particularly useful, helping patients follow exercises more consistently: “They seem to adhere a lot better to it when the phone pings at them and tells them to do it” (Physiotherapist 4). Similarly, goal-setting features were appreciated for creating clear expectations and providing structure: “Setting the goals ... is a very good, nice incentive for the patient” (Surgeon 1).

Clinicians also highlighted how progress tracking helped patients maintain perspective during recovery. One physiotherapist noted that visualizing change over time enabled more constructive conversations: “It’s really nice to be able to say, ‘But look how you were here’ ... you actually forget quite how bad you were at the start” (Physiotherapist 3).

### Challenges or Weaknesses

#### Overview

**Figure 4.**
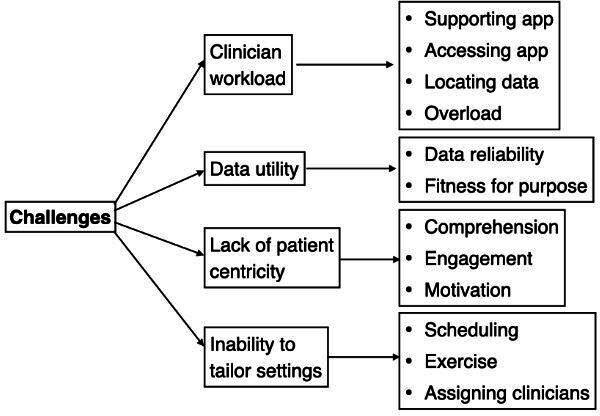
Reported challenges.

Despite the benefits noted in the previous section, there were also many noted shortcomings, which draw attention to the ongoing challenges in designing and implementing PGHD-inclusive software. In the following sections, we discuss ways in which the software was reported to often increase clinician workload, data utility was often questionable, was ill-suited for patient needs, and the software lacked the flexibility to be tailored to clinical workflows (Figure 4).

#### Clinician Workload or Time

Clinicians reported that the app introduced additional workload and usability challenges. For example, managing patient queries about technical issues, such as step-count tracking, was time-consuming (Physiotherapist 4). Accessing the platform also required logging into multiple systems, which several participants described as cumbersome or unrealistic within the constraints of outpatient care: “It’s too difficult to log on to lots of different things” (Physiotherapist 5).

Beyond accessibility, participants expressed frustration with the fragmentation and volume of data, which often lacked clinical clarity. One surgeon noted, “Too much information and not quite sure what the information it is giving me is ... I don’t even know what double support percentage means” (Surgeon 2). Others echoed this sentiment, describing the system as cognitively overwhelming and poorly integrated into existing workflows. As one clinician put it, “It’s a lot of data” (Physiotherapist 1).

#### Utility

Beyond issues of access and cognitive load, clinicians expressed doubt about the reliability and clinical utility of patient-generated data. Some were concerned that behavioral data (eg, step counts) lacked the necessary context. As one physiotherapist explained, “If someone’s doing 3000 steps but limping badly and in significant pain, that’s not particularly useful for us” (Physiotherapist 4). Others questioned the accuracy of passive data collection, noting that reduced activity might reflect patients not carrying their phones, rather than a true change in function.

Several participants also highlighted the insufficiency of quantitative metrics alone, emphasizing the continued need for direct clinical assessment: “You have to look at the patient, you can’t just look at the data” (Physiotherapist 2). For many, the data offered limited actionable value, and some felt it had minimal influence on therapeutic decision-making. As one participant concluded, “It doesn’t change our practice in any way, and it almost makes it longer” (Physiotherapist 4).

#### Lack of Patient Centricity

Clinicians emphasized that the app’s design did not fully account for the cognitive, motivational, and contextual limitations faced by many patients. Some described difficulties with patients who struggled to understand what was being asked of them, especially those with cognitive impairments or who were frail and living in care settings. As one clinician put it, “A lot of the patients we see here are also quite frail, quite elderly ... I think it may just be one too many things for them to manage” (Surgeon 4). Another noted that misunderstandings about the purpose or expectations of PGHD were common: “One of the biggest challenges is everyone’s understanding of exactly what’s being asked of them and how they measure that accurately” (Physiotherapist 5).

In addition to these accessibility issues, clinicians also reflected on the unintended psychological effects of goal-based metrics and countdowns. While some patients found feedback motivating, others reacted negatively to perceived regressions or delays. “If they see they’re slipping back,” one physiotherapist explained, “then it’s that defeat ... ‘What’s the point?’” (Physiotherapist 3).

#### Inability to Tailor to Patient Journey

Clinicians expressed frustration with the app’s limited flexibility to accommodate real-world variations in patient pathways and care team structures. One key issue was the fixed countdown timer, which could become inaccurate after surgery and block access to relevant content: “Her app is now telling her it’s 52 days to her surgery ... but she’s just had it” (Physiotherapist 3). This lack of adaptability also extended to exercise programming. Physiotherapists reported that postoperative exercise options were too generic to meet the diverse needs of patients and could not be tailored to individual recovery trajectories. As one noted, “There’s only two programs ... some exercises are too easy for certain patients, some are too difficult ... there’s no way of changing them” (Physiotherapist 5). In some cases, clinicians advised patients to ignore or delete the app in favor of more appropriate alternatives.

In addition to content rigidity, participants also pointed to deficiencies in role-based functionality. They reported being unable to assign specific clinicians to patients, which led to communication breakdowns and unclear responsibilities. “There’s no way of us assigning ourselves to that patient on the app,” one physiotherapist explained, “so they may still get pinged with exercises we’re not using” (Physiotherapist 5). Surgeons likewise described limited visibility into patient activity between referral and surgery, undermining continuity of care (Surgeon 4).

### Opportunities for Improvement

#### Overview

**Figure 5.**
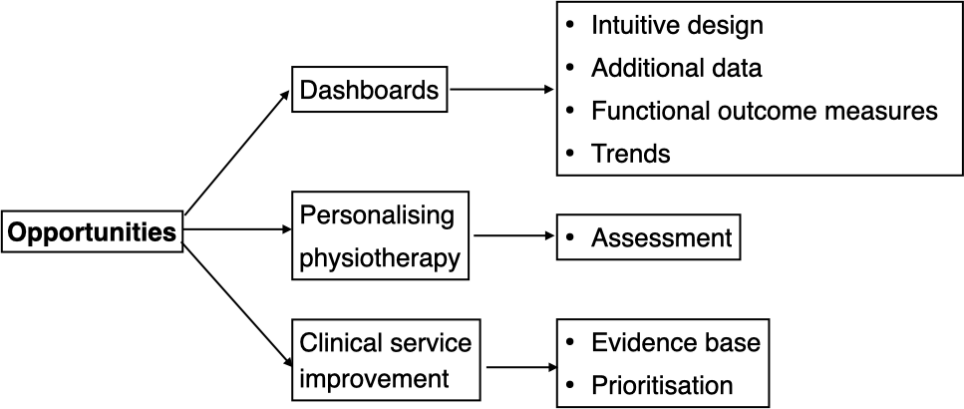
Opportunities.

As well as the benefits and shortcomings noted in the previous section, our analysis suggests several opportunities for enhancing future systems in dashboard design, personalization of physiotherapy, and clinical service improvement. Participants suggested making dashboards more intuitive and informative, with real-time notifications and interest in adding additional data such as heart rate and pain levels. They also emphasized the need for trend identification in patient data to better track progress over time. Personalizing physiotherapy was highlighted as crucial, with tailored exercises based on individual weaknesses and goals, especially after the operation. Finally, the potential of PGHD to improve clinical care and surgical prioritization was recognized, suggesting that more detailed and personalized patient data might enhance treatment outcomes and resource management (Figure 5).

#### Dashboard Design

Clinicians proposed several enhancements to dashboard design and data presentation. Many expressed a desire for a more intuitive overview of patients with real-time updates and alerts for significant changes. One surgeon asked, “Why can’t I have [notifications] for my patients?” and described wanting a sidebar dashboard to monitor patient status more easily (Surgeon 2). Participants also suggested visual elements like color coding to draw attention to significant changes in patient mobility (Surgeon 3).

Requests for additional data included heart rate and pain levels, especially during the early recovery phase. “Pain is usually the really big indicator ... in that first six-week period,” noted one physiotherapist (Physiotherapist 4). Others emphasized the importance of context—knowing not just pain scores, but how they relate to exercises, walking aid use, and movement patterns (Physiotherapist 5).

There was strong support for capturing functional outcome measures that reflect daily living. Participants emphasized activities like dressing, standing from a chair, driving, or using public transport as better indicators of recovery than static scores. As one clinician put it, “It’s all about their ability to get on with daily life” (Physiotherapist 3).

Finally, clinicians stressed the value of visualizing patient trends over time. One surgeon explained that even if absolute scores remain low, improvements in mobility or independence may still represent substantial recovery: “Trend is more important than absolute” (Surgeon 4). Another participant noted that having longitudinal data could significantly improve follow-up consultations after long gaps (Physiotherapist 3).

#### Personalizing Physiotherapy

Clinicians highlighted the need to personalize physiotherapy plans based on individual patient characteristics, especially postoperatively. “I give different exercises to most of my patients depending on where their weakness is, where their limitation in movement is, and what their goals are,” explained one physiotherapist (Physiotherapist 5). Others emphasized that while protocol-based rehabilitation may suffice preoperatively, postoperative recovery is highly variable. “After the operation, so much depends on what changes ... everyone’s different,” noted Physiotherapist 4.

#### Clinical Service Improvement

Participants saw potential for PGHD to improve clinical services by informing population-level trends, surgical practices, and prioritization processes. One surgeon noted that, with sufficient data, PGHD could help identify common rehabilitation challenges or communication barriers and enable more targeted support: “We can look into what works for most of the patients ... and target those patients specifically” (Surgeon 1). Others reflected on how such data could prompt critical self-assessment of surgical decisions—for example, identifying stiffness patterns that might relate to prosthetic fit (Surgeon 4).

PGHD were also seen as a tool for refining surgical prioritization. “If their mobility is decreasing rapidly, that’s very important ... so we can try to get them in and have their operation quicker” (Surgeon 1). The dynamic use of deterioration data to rerank patients on waiting lists was described as a promising but underused opportunity.

## Discussion

### Principal Findings

In this section, we discuss the benefits, challenges, and opportunities raised through our analysis. First, we examine the software’s potential to streamline patient education, improve communication, and enhance motivation. We then explore the hurdles of increased clinician workload, clinician concerns about the reliability or credibility of patient-submitted data, and patient engagement variability. We finally discuss emergent tensions and propose further research to help resolve ongoing challenges.

### Benefits

The interviews suggested that the mymobility software with PGHD integration could offer meaningful benefits in the orthopedic care setting. Structured education plans and essential information were appreciated for reducing staff workload and helping patients to prepare for operations. The communication and data sharing functions were useful for monitoring patients and, in some cases, helping to inform interventions. The use of reminders, goal setting, and the ability for patients to view progress over time were noted as showing promise for motivating patients to follow pre- and postoperative care plans. Perhaps most promising for asserting the value of PGHD integration was Surgeon 1 noting that there were specific cases where the shared data had provided evidence for prioritizing care. However, it is important to note that app-based education and preoperative physiotherapy support, some of the more successful noted aspects of the app, do not necessarily require PGHD integration. It remains for further research to determine if PGHD could help with further personalization, for example, with support for comorbidities [32], and if in these cases, benefits would outweigh operational challenges. Physiotherapist 4 noted that the great variance in postoperative outcomes requires in-depth knowledge of the patient’s abilities and progress, which could potentially be supported by greater PGHD integration, though the interviewees expressed concerns that this was more reliably and with less effort accomplished through in-person contact (Physiotherapist 4 and Physiotherapist 2). It is noteworthy and promising that many of the potential benefits of PGHD cited in the literature review were to at least some degree reflected in these interviews and in some cases delivered by the software. As per Cohen et al [4], PGHD did help to provide insights; as per West et al [5], it did help to facilitate care personalization; and as per Caldeira et al [9], the collected data showed promise in motivating and engaging patients. While these benefits might not be universally reported by all clinicians, the responses suggest that this software is making strides in delivering the promised benefits of PGHD integration, and this study provides some empirical evidence that it is viable to integrate PGHD within orthopedic clinical practice.

### Challenges

Despite the noted benefits, clinicians reported many barriers to integrating PGHD within clinical care, often echoing the known barriers cited in our review. Particularly problematic were the many ways the app could increase clinician workload, from requiring time to support patients in using the app (Physiotherapist 4), interacting with the platform (Surgeon 2), and locating and making use of data (Physiotherapist 5). Potential benefits of the platform were also complicated by the clinicians’ continued reluctance to trust the validity of PGHD (Physiotherapist 4) and challenges in applying data to decision-making (Physiotherapist 2). Furthermore, clinicians expressed concerns that the need for patients to participate with this approach was unsuitable for many of their patients, echoing the study by Jim et al [6], which noted that the use of PGHD in treatment could increase care disparities. The interviews also highlighted the challenges in tailoring software to reflect individual needs, such as the selection of postoperative exercises or the assigning of clinicians. While the noted interface design shortcomings are likely correctable, the inability to overcome the larger barriers cited within the review is concerning, given the resources likely invested in this software’s design and development. In addition, defining the requirements for the many configurations needed to adequately address personalization of the patient journey likely remains a stubborn problem [33]. This is especially vital, as clinicians noted how this inability to properly reflect the individual patient journey can easily lead to software rejection. Similarly, this leads to another broader question as to how many features should be included within PGHD systems, given how a single deficiency area such as improper notifications can lead to product abandonment.

### Opportunities

Clinicians suggested many realizable and relatively standard suggestions for improving product usability, such as making interface terms easier to understand, expanded use of color coding, and indications of how patient progress deviates from norms or expectations. There were also calls for increasing convenience through new features such as notifications of important events and streamlining sign-ins, as well as potentially integrating additional data sources like heart rate, range of movement, pain levels, gait patterns, and increased integration of functional outcome measures to support a more holistic understanding of patients’ needs. Such suggestions appear relatively straightforward and reflect established design practices and features within other products. However, other suggestions, such as automated exercise adaptation to individual patient need, might pose greater challenges, given that the clinicians emphasized the importance of human interaction in making these adjustments. While the use of PGHD for clinical service improvement appears especially promising, it might require standardization and trust in PGHD, which might still be culturally lacking at this time.

### Design Tensions for Further Research

The software demonstrated capacity to deliver value to clinical care, and many of the noted design deficiencies and suggested improvements are likely amenable to improvement through established iterative human-centered or co-design techniques. Other deficiencies relate to broader societal concerns, which are outside of the ability of software systems to address. In this section, we identify key areas for further research that consist of trade-offs or tensions that might imply conflicting requirements, which cannot be “solved” but rather require design decisions that recognize that trade-offs are inherent. While not necessarily novel, their persistence in this software after significant industry investment suggests that software design alone is insufficient to drive PGHD into the mainstream.

#### Leveraging Bigger Data Versus Burdening Clinicians

Participants were impressed with the range of available data presented within the mymobility app and even suggested adding additional data. However, they also noted that expansive data could be overwhelming, could increase workload, and that they were often unsure of how to apply such data to their practice. Research question: How can PGHD dashboards be designed to decrease clinician workload? How can the system deliver the right data at the right time to the right clinical role?

#### Standardization Versus Personalization in Clinical Practice

While standardized protocols and data collection methods are necessary for consistency and reliability, clinicians emphasized the importance of personalized care, particularly in postoperative physiotherapy, where individual patient needs vary significantly. Research question: How can PGHD systems balance the need for standardized data collection and protocols with the flexibility required to personalize patient care effectively?

#### Comprehensive Versus Targeted Software

Study participants related challenges resulting from accessing information and functionalities from multiple systems, which could support the need for a single comprehensive approach. Yet, participants also noted how one inappropriate feature could lead to system rejection. Research question: What is the appropriate balance in the number of features to include within PGHD-inclusive orthopedic software? How should this be determined?

#### Adaptive Versus Static Design Approaches

It is recognized that health care is complex with outcomes emerging from multiple interacting systems needing frequent adaption to shifting contexts [34]. This creates a tension between the validation needed to gain regulatory approval for clinical software systems and the dynamic requirements within clinical contexts. Research question: How can clinical software systems be designed that can be validated yet are sufficiently adaptable to variable workflows, preferences, and evolving needs?

### Limitations

This study has several limitations. The sample size was limited due to real-world constraints during the COVID-19 pandemic, including staffing shortages and scheduling disruptions. Although we sought diverse perspectives, these logistical challenges prevented broader recruitment and may have introduced selection bias. Additionally, recruitment was limited to a single orthopedic center, which may constrain the transferability of findings. This was due to institutional access constraints and pandemic-related limitations. Future research should explore multicenter participation to capture a broader range of experiences with PGHD integration. Recruitment methods using snowball sampling and existing professional relationships may also introduce selection bias, and the reliance on self-reported data could affect accuracy due to recall bias or socially desirable responses. Additionally, the varying levels of technological familiarity among clinicians and the unique circumstances of the COVID-19 pandemic, which increased reliance on digital tools, may have influenced the results. Future research should address these limitations by including a larger, more diverse sample across multiple health care settings and ideally using random sampling methods to better capture the evolving nature of PGHD integration in clinical practice. Furthermore, as the study was limited to clinicians working with patients who have had TKA or THA operations, the findings may not be transferable to other orthopedic contexts such as trauma, spine, or sports medicine. Future research should investigate the applicability and value of PGHD integration across a wider range of orthopedic conditions.

### Conclusions

This study sought to close gaps in knowledge about the real-world application of PGHD integrative software within orthopedic practice. To this end, surgeons and physiotherapists were interviewed to learn more about their experiences and suggestions for improvement. The findings suggest that the mymobility software delivered some meaningful benefits. Structured education plans and the providing of essential information were praised for reducing staff workload and helping patients prepare for operations. The communication and data-sharing functions showed promise for monitoring patients and informing interventions. Additionally, reminders, goal setting, and progress-tracking features showed the capacity to motivate patients to adhere to pre- and postoperative care plans. Notably, the integration of PGHD helped prioritize care in specific cases, indicating its potential in enhancing clinical decision-making. While some benefits, such as app-based education and preoperative physiotherapy support, do not necessarily require PGHD integration, the software still aligns with literature-reviewed benefits, providing insights, facilitating personalized care, and motivating patients. While there remained a clear need for further interface and interaction design, the software appears to be making strides in delivering on the promised benefits of PGHD within a clinical context.

However, many of the known barriers to PGHD integration remain unresolved. Clinicians reported challenges such as the app increasing their workload by requiring time to support patients in using the app, interacting with the platform, and locating and making use of data. Potential benefits were further hampered by clinicians’ reluctance to trust the validity of PGHD and challenges in applying data to decision-making. Additionally, the patient participation required for a PGHD approach posed significant challenges, especially for those who are older or have cognitive impairments. Although the mymobility platform has already been scaled commercially, this study shows that availability does not guarantee seamless clinical integration. Participants described persistent barriers—such as limited time to engage with PGHD, doubts about its clinical relevance, and challenges supporting diverse patient populations—that complicated effective use in practice. These findings suggest that even at scale, PGHD platforms require ongoing refinement of interface design, clinician workflows, and support strategies to ensure meaningful and sustainable adoption.

While many of the perceived benefits and barriers align with prior findings from similar digital health interventions, this study adds value by contextualizing those dynamics within the day-to-day realities of orthopedic care. The role-specific insights from surgeons and physiotherapists offer a practical understanding of how PGHD are actually interpreted, adapted, and applied within clinical workflows.

Future research should explore how to address trade-offs or tensions that may not be fully “solvable” but instead require thoughtful design decisions. Research and development efforts should aim to navigate these complexities in ways that improve usability, utility, and adoption. Further studies should also build on these findings by using quantitative or mixed methods approaches to evaluate the measurable impact of PGHD on clinical workflows, patient engagement, and health outcomes.

## Abbreviations

PGHD: patient-generated health data
RTA: reflexive thematic analysis
STSD: sociotechnical systems design
THA: total hip arthroplasty
TKA: total knee arthroplasty
